# Necessity of More Heroic Treatment in Cases of Lumbar Abcess

**Published:** 1878-10

**Authors:** Edmund Andrews

**Affiliations:** Professor of Surgery in the Chicago Medical College; No. 6 Sixteenth St.


### THE NECESSITY OF MORE HEROIC TREATMENT IN CASES OF LUMBAR ABSCESS.

BY EDMUND ANDREWS, A. M., M. D.

*(Professor of Surgery in the Chicago Medical College.)*

The condition of surgical literature on the subject of lumbar abscess is a disgrace to science. Saying nothing of spinal disease, a frequent cause of lumbar abscess, in discussing which our standard authors show an astounding ignorance of the present state of orthopedic surgery, let us pass on to the operative treatment of the abscesses themselves. In this department the surgical authorities are in utter and hopeless contradiction, and what is worse, where they find themselves destitute of a solid scientific basis for their opinions, many of them have not the manliness to acknowledge the situation, but emit a perfect fog of loose and random opinions to cover their ignorance.

Science above all things should be candid and truthful. When shall we have a body of savants who will not only state what they know, but will in humility and honesty acknowledge what they do not know?

In respect to the opening of lumbar abscesses, Sir Astley Cooper advised to evacuate them with valvular incisions from time to time, keeping them carefully closed between the operations. This was to avoid the decomposition of the pus with the consequent hectic and pyæmia which follows so frequently where the incision is left open. Chelius and Lawrence agreed with Sir Astley, Dupuytren advised not to open them at all, but to let them break spontaneously. Abernethy advocated the plan of valvular incision. J. S. South advised to wait until the abscesses were just about to burst spontaneously, and then to open them with a small puncture, leaving them open for gradual evacuation.

Gross declares that these abscesses all spring from carious vertebræ, contrary to the opinion of the rest of the profession. He says there is little or no hope of cure or benefit by any known local surgery, and therefore he very honestly omits to recommend any. He has no faith in antiseptic plans.

Erichsen says, wait until the abscess is about to burst spontaneously, and then evacuate it either by a valvular incision, by aspiration, or by Lister's antiseptic plan.

Ashurst, of the Episcopal Hospital, Philadelphia, says, do not open them at all, but let them burst spontaneously; but if you insist on opening them, use Lister's antiseptic plan.

Bryant, of London, says, open early and freely on the antiseptic plan.

Gant, of the Royal Free Hospital, London, advises to wait until the abscess is ready to burst, and then to evacuate it with a valvular incision, and close it again.

Frank Hamilton, of New York, directs first to invigorate the general health with tonic treatment and then to open the abscess, but to use no antiseptic injections, drainage tubes, tents nor even probes; at least not until it has been discharging for some weeks.

Callender, of St. Bartholomew's Hospital, London, says, open early, insert drainage tubes as far in as possible, and inject antiseptics at once, using pressure to make the fluid penetrate the remote parts of the sac.

Holmes, of England says, allow the abscess to burst spontaneously.

Heath and Owen, of London, direct to open it, insert a drainage tube, and use antiseptic injections under pressure.

Druitt counsels opening from time to time with small punctures and reclosing them.

Lister, late of Glasgow, now of Edinburgh, advises to open the abscess and to use drainage tubes, with antiseptic injections and dressings.

From this melange of contradictions the practical physician and surgeon is expected to evolve a rational plan of treatment, and it is no wonder that some, like Prof. Gross, give it up as an insoluble conundrum.

But there is no need of this confusion of ideas. Some truths in surgery are irrevocably settled and they are just as applicable to lumbar abscess as to similar collections of pus elsewhere, only they need a little more heroic energy in their use.

First. We all know that large collections of pus undergo a disastrous putrefaction if they are opened to the external air, and that hectic, fever and fatal pyæmia are prone to follow at once.

Second. If this opening is made so as not to admit the septic germs of the atmosphere, or if antiseptic injections and dressings are faithfully used, the evacuation is usually free from danger.

Third. Drainage tubes, when efficiently used, prevent the contraction of the outlet, allow free evacuation and admit of efficient antiseptic injection, and when such injections can be made to bathe the whole interior of an abscess, they will almost invariably stop the suppuration with its whole train of disasters.

Callender, of London, recognizes the truth of these principles and has applied them to some extent to lumbar abscesses, as have also, Heath, Lyster and Owen.

There are peculiar difficulties of a mechanical nature to be overcome in these vast abdominal collections of pus, which now demand our attention, and which, as I maintain, will often require more heroic treatment than has hitherto been brought to the attack.

The abscesses under consideration, commence in the vicinity of the psoas muscles, sometimes in carious vertabræ, and sometimes in the loose connective tissue of the vicinity without any bone disease. Hence they are often called psoas abscesses, or if they commence in the iliac fossa, iliac abscesses. Wherever they originate, they may extend both upward and downward, and at last break externally. In most instances they point either above in the lumbar region, or below in the front half of the thigh, passing under Poupart's ligament. There is no definite rule deciding which direction will be taken, and some abscesses point both above and below simultaneously. The cavity is vast in size and irregular in form, but it is conspicuously divided into two parts, viz.: the internal sac, contained within the abdominal walls, and the external sac which spreads out between the skin and the deep fascia. The internal sac discharges into the external one through a long, crooked and narrow outlet.

Now two difficulties have hitherto beset the treatment of these cavities by antiseptic injections.

First, the liquid thrown in is prone to fill only the external sac, and to be so resisted by the long, narrow fistula, blocked with pus, which leads to the inner cavity, that it fails to reach the far off interior, and consequently arrests neither the suppuration nor the decomposition. It is to meet this difficulty that Callender, and a few other English surgeons lately advocate what some of us have already practiced a considerable time in Chicago—viz., the use of injections forced in under pressure, in the hope of thus reaching the interior recesses. The other difficulty is that both the opening in the skin, and the fistula of connection between the two sacs are prone to contract, so as to resist equally the evacuation of the abscess and the injection of antiseptics.

Both here and in London, we have endeavored to counteract this by the drainage tube, but too often could only insert it into the external orifice, while the internal fistula of communication was either wholly missed, or only tubed through a part of its length. It is just here that I wish to advocate more heroic measures. I think the internal sac should be reached at all hazards, or if that is too strong a term, at some hazard, for if it is not done, the patient will have to meet a terrible increase of danger. If the abscess points in the lumbar region, the operation is not seriously difficult. After reducing the size of the cavity by a suitable number of preliminary tappings, the external sac, which is only covered with skin and fat, should be freely opened and its interior searched with a sound or catheter to find the fistula leading inward. If there is difficulty, the incision may be prolonged upward as far as necessary. When the route is discovered, a drainage tube must be carried well into the interior of the body, and made the route of copious, and if need be, forced injections.

If the pointing of the abscess is below Poupart's ligament, the difficulty is greatly enhanced, but the principles of the operation are the same. The external sac ought, in my opinion, to be laid open to any extent necessary to enable the surgeon to find the upward route, and then measures should be taken to pass a flexible drainage tube far up into the abdominal part of the abscess. In old contracted cases I have sometimes found it impossible to accomplish this. Now if the patient in such case is found to be steadily running down in health, he will be, I think, justified in running some risk, since he has little ground of hope in any other way. It would be obviously possible to first make a dissection below Poupart's ligament and finding the fistula, pass a tube as far up into the iliac fossa as possible; then we could make an incision above the ligament in the way we do when tying the iliac artery, and carefully lifting the peritoneum from the iliac muscle, we shall soon feel and reach the tube inserted from below. Now we can open and enlarge the fistula upward and place a drainage tube quite up into the lumbar region, and bring out its lower end above Poupart's ligament. If this route in any case is thought unadvisable, I would advocate cutting in at the lumbar region as in lumbar colotomy, only keeping very close to the quadratus lumborum muscle, and following its inner surface inward to the psoas, cautiously lifting up the peritoneum until the abscess is found. In case of difficulty in finding the cavity of pus, exploration with the aspirator would often show the route. Once found, the abscess could be furnished with a drainage tube running straight outward, and placed in conditions well adapted to effect a cure.

Of course I do not advise to subject every patient to this bold treatment. The proper course is clearly this.

As soon as the abscess points at all externally, it should be aspirated, running the needle in obliquely in the thicker portion of the sac, so as to ensure re-closure. To prevent septic results, the needle and the skin should first be carbolized. When the cavity is partly filled again, it should be aspirated a second, and if necessary a dozen times.

In this way no perceptible risk is run, and in many cases the pus will be found gradually becoming more serous, and showing fewer corpuscles, until at length it is almost as transparent as water, and the abscess is gradually obliterated without ever having been open to the air; meanwhile the patient gets fat and rosy, and shows none of the dangerous symptoms resulting from open air discharges. If the pus continues to form too persistently, the cavity can be washed out at the time of each tapping with carbolized water.

However there are two circumstances which may prevent success. First, the puncture of the aspirator needle may fail to heal, and commence some day to discharge. This accident is frequent, and in expectation of it the patient should be provided beforehand with the means of instantly making a carbolized injection and dressing without waiting for the surgeon. The other obstacle to success is that the pus may be filled with curdy concretions, so that it will not run through an aspirator, nor even through a large trochar. In this case I advise a free valvular incision, and the evacuation of clots and all through it, with an effort at re-closure. If this effort fails, I would have prompt recourse to a drainage tube, with any incisions and operations which may be found necessary as above stated, to insert the tube well within the inner sac.

I have not yet been obliged to make the elaborate pelvic dissection from below, but I have tubed the inner sac three times from the lumbar region, twice with complete success, the patients appearing now out of danger. The third case was a remarkable one, for the abscess pointed in both groins, and in the lumbar region, discharging from all three points at once.

I tubed the inner sac through the lumbar route, and apparently for some months arrested the downward progress of the patient; however she resided in quarters which were foul with putrid air, and which had no possibility of ventilation. She died at last of an attack of accute gastro-enteritis.

In conclusion, I would urge again the importance of early and energetic surgical action, and, if the preliminary measures fail, the resort to bold operative measures to enable us to drain and inject the internal sac of the abscess.

No. 6 Sixteenth St., Sept. 2, 1878.

